# Enhancing atrial‐specific gene expression using a calsequestrin *cis‐*regulatory module *4* with a sarcolipin promoter

**DOI:** 10.1002/jgm.3060

**Published:** 2018-12-04

**Authors:** Jimeen Yoo, Erik Kohlbrenner, Okkil Kim, Roger J. Hajjar, Dongtak Jeong

**Affiliations:** ^1^ Cardiovascular Research Center, Icahn School of Medicine at Mount Sinai New York NY USA

**Keywords:** AAV9, atrium, *cis*‐acting regulatory module, *CRM4*, gene therapy, sarcolipin

## Abstract

**Background:**

Cardiac gene therapy using the adeno‐associated virus serotype 9 vector is widely used because of its efficient transduction. However, the promoters used to drive expression often cause off‐target localization. To overcome this, studies have applied cardiac‐specific promoters, although expression is debilitated compared to that of ubiquitous promoters. To address these issues in the context of atrial‐specific gene expression, an enhancer calsequestrin *cis‐*regulatory module *4* (*CRM4*) and the highly atrial‐specific promoter sarcolipin were combined to enhance expression and minimize off tissue expression.

**Methods:**

To observe expression and bio‐distribution, constructs were generated using two different reporter genes: luciferase and enhanced green fluorescent protein (EGFP). The ubiquitous cytomegalovirus (CMV), sarcolipin (SLN) and *CRM4* combined with sarcolipin (*CRM4*.SLN) were compared and analyzed using the luciferase assay, western blotting, a quantitative polymerase chain reaction and fluorescence imaging.

**Results:**

The CMV promoter containing vectors showed the strongest expression *in vitro* and *in vivo*. However, the module SLN combination showed enhanced atrial expression and a minimized off‐target effect even when compared with the individual SLN promoter.

**Conclusions:**

For gene therapy involving atrial gene transfer, the *CRM4*.SLN combination is a promising alternative to the use of the CMV promoter. *CRM4*.SLN had significant atrial expression and minimized extra‐atrial expression.

## 1 INTRODUCTION

Cardiac diseases are the leading cause of death but treatment options are still inadequate. Cardiac gene therapy has emerged as a promising method for combating cardiac diseases. Currently, adeno‐associated virus serotype 9 (AAV9) is the most promising delivery vehicle for transgene expression and the most widely used in cardiac preclinical trials because of its tropism for the heart.^1^, ^2^, ^3^, ^4^ Although the AAV9 vector in conjunction with the ubiquitous cytomegalovirus (CMV) promoter has shown cardiac specificity, a major limitation is the expression of transgenes in other organs, such as liver, lung, thymus, brain and kidney.^5^, ^6^, ^7^ Concerns with respect to tissue specificity have been managed previously through transcriptional targeting.^8^ Because the virus regulates transgene production through its promoter, promoter alterations have been used to control gene expression in various organs to avoid off‐target effects. To overcome this barrier, cardiac‐specific promoters have been applied.

Current cardiac‐specific promoters investigated include α‐myosin heavy chain, myosin light chain, enhanced myosin light chain and cardiac troponin T promoters.^9^, ^10^, ^11^, ^12^, ^13^, ^14^ Among the promoters investigated, some have shown chamber specificity. Because the atrium and ventricle differ morphologically, functionally and are molecularly distinct, pathophysiology also varies in cardiac diseases. Thus, using a cardiac‐specific promoter for universal transgene expression in the heart in certain disease states can also affect healthy tissues negatively. Accordingly, the use of a specific promoter is critical in chamber‐specific diseases.

Currently, several promoters have been observed to regulate cardiac chamber‐specific gene expression. Among these are the atrial myosin light chain‐2a, slow myosin heavy chain‐3, atrial natriuretic factor and sarcolipin (SLN) for the atrium and the myosin light chain‐2V for the ventricle.^15^ The uniqueness of these promoters has been exploited in other methods in addition to improving specificity, such as allowing for cardiomyocytes generated from human‐induced pluripotent stem cells to be separated by subtype‐specific promoter‐driven action potentials.^16^ However, the application of these promoters to drive chamber‐specific transgene expression has been limited as a result of the compromised efficiency of these promoters. To apply these promoters for use in gene therapy, the efficient transduction of these promoters is essential.

To overcome this, two sequences established previously were used to make an atrial‐specific promoter with enhanced expression. We approached this issue by taking advantage the atrial‐specific promoter of SLN. The SLN promoter was chosen because the SLN protein is characteristically expressed in the atrial chamber of the heart. Furthermore, it has been observed to change dependent on disease states. Sarcolipin was found to be up‐regulated in rodent models of congenital heart disease and in patients with preserved left ventricular ejection fraction and chronic isolated mitral regurgitation.^17^, ^18^ By contrast, SLN was found to be down‐regulated in patients with chronic atrial fibrillation.^19^ In Duchenne muscular dystrophy mice, a reduction in SLN expression was found to mitigate associated cardiomyopathy.^20^

Alone, the SLN promoter activity is unsubstantial compared to that of ubiquitous promoters. Thus, the addition of calsequestrin 2 *cis‐*regulatory module *4* (*CRM4*), a cardiomyocyte‐specific enhancer, which showed superior activity in the heart, was used to heighten transgene expression of SLN within the heart.^21^

In addition to cardiac tissue, high SLN levels are found in the skeletal muscle and diaphragm.^22^, ^23^ The addition of *CRM4* was thus hypothesized to improve cardiac specificity. When used in conjunction with the cardiotropic AAV9, we hypothesized that there would be even higher selectivity in the heart.

The bio‐distribution of an AAV9 vector expressing two reporter genes [luciferase or enhanced green fluorescent protein (EGFP)] under the control of the CMV, SLN or the *CRM4* and SLN combination promoters was evaluated. The present study investigated whether an adeno‐associated viral vector driven by an enhanced SLN promoter results in specific and improved transgene expression in the atrium.

## 2 MATERIALS AND METHODS

### 2.1 Promoter construction

#### 2.1.1 pTR.CMV.EGFP

This vector was kindly provided by Dr R. J. Samulski (University of North Carolina, Chapel Hill, NC, USA).

#### 2.1.2 pTR.CMV.Luc

This vector was kindly provided by Dr R. J. Samulski (University of North Carolina).

#### 2.1.3 pTR.SLN.Luc

The shortened SLN promoter used was 1029 bp. This was identified as the primary core promoter sequence by removal of polyadenylation sequence from a 1253‐bp human SLN promoter purchased from GeneCopoeia (HPRM12771; GeneCopoeia, Rockville, MD, USA). SLN primers were generated as: forward: 5′‐CCTAGATCTGAATTCGGTACCTGAGGAATGGGA‐3′ and reverse: 5′‐CGGTGTGCCTCTCATACCGGTTCTGCCTTTCTCATT‐3′. The SLN promoter region with *Kpn*I and *Age*I enzyme sites was amplified using a polymerase chain reaction (PCR). pTR‐CMV‐Luciferase digestion with *Kpn*I and *Age*I removed the CMV promoter sequence. The promoter and vector sequences were ligated to create the pTR‐SLN‐Luciferase vector.

#### 2.1.4 pTR.SLN.EGFP

*CMV.EGFP* was digested with *Kpn*I and *Age*I to remove the CMV promoter sequence. The amplified SLN promoter used to construct *SLN.Luc* was used here. The promoter and vector sequences were ligated to create the *SLN.Luc* vector.

#### 2.1.5 pTR.*CRM4*.SLN.Luc

The 1029‐bp SLN promoter was PCR amplified from the pTR‐SLN‐Luciferase with primers: forward 5′‐GAGCAAACACAATTGCTAGGG‐3′ and reverse 5′‐AAGAGGATCAAAGACACACC‐3′. A second PCR amplification was performed to add Gibson assembly overhangs: forward 5′‐GATACAGTCTGTCCGAACGCGTGGAGCAAACACAATTGCTAGGG‐3′ and reverse 5′‐ACAGTACCGGAATGCCAAAGAGGATCAAAGACACACC‐3′. The *CRM4* sequence was obtained from Dr T. VandenDriessche (University of Brussels, Brussels, Belgium). A parental plasmid containing previously cloned *CRM4*‐Luciferase was digested with *Mlu*I and *Hind*II to insert the SLN promoter with overhangs between the *CRM4* and luciferase using the GeneArt Seamless Assembly kit (Thermo Fisher, Carlsbad, CA, USA).

#### 2.1.6 pTR.*CRM4*.SLN.EGFP

The above pTR‐ESG was digested with *Nco*I and *Eco*521 to remove the luciferase gene. The *CMV.EGFP* shuttle vector obtained from Dr R. J. Samulski (University of North Carolina) was digested with *Nco*I and *Eco*521 to remove EGFP. The luciferase gene was replaced with the EGFP gene digested from plasmid *CMV.EGFP*.

### 2.2 Generation of AAV

The following self‐complementary AAV (serotype 9) constructs were generated: *CMV.Luc*, *CMV.EGFP*, *SLN.Luc*, *SLN.EGFP*, *CRM4.SLN.Luc* and *CRM4.SLN.EGFP* vector. The recombinant AAV was produced by transfecting HEK293‐T cells (ATCC, Manassas, VA, USA) as described previously.^24^ The AAV particles in the cell culture media were collected by precipitation with ammonium sulfate and purified by ultracentrifugation on an Optiprep iodixanol gradient (Sigma‐Aldrich, St Louis, MO, USA). The particles were concentrated by exchanging iodixanol for Lactate Ringer's solution (Baxter Healthcare Corporation, Deerfield, IL, USA) by multiple dilution and concentration steps using a Vivaspin 20 Centrifugal concentrator 100 K MWCO (Sigma‐Aldrich). The AAV titer was determined by quantitative real‐time PCR (qRT‐PCR) and sodium dodecylsulfate‐polyacrylamide gel electrophoresis (SDS‐PAGE).

### 2.3 *In vitro* gene transfer

Cardiac muscle cell line, HL‐1, was maintained in Claycomb medium (Sigma‐Aldrich) supplemented with 10% fetal bovine serum (Sigma‐Aldrich), penicillin (100 U mL^–1^), streptomycin (100 U mL^–1^), 2 mM l‐glutamine, 0.1 mM noradrenaline (Sigma‐Aldrich) and passaged approximately every 3–4 days until cells reached confluency and spontaneous contractions were observed. Cells were transfected with *CMV.VLP*, *CMV.Luc*, *SLN.Luc* and *CRM4.SLN.Luc* viral vectors. After an additional 24 hours of incubation, cells were harvested and used for the luciferase assay.

### 2.4 Animal care and *in vivo* gene transfer

All procedures were approved by and performed in accordance with the Institutional Animal Care and Use Committee of the Icahn School of Medicine at Mount Sinai. The investigation conforms with the Guide for the Care and Use of Laboratory Animals (NIH Publication No. 85–23, revised 1996). Studies were conducted in male B6C3F1 mice aged 8–10 weeks (weight 25–30 g) obtained from Jackson Laboratories (Bar Habor, ME, USA). *In vivo* gene transfer was performed by immobilizing mice with a single mouse restrainer (Harvard Apparatus, Cambridge, MA, USA) and 100 μL of viral solution (1 × 10^11^ vg or 5 × 10^11^ vg) of the pertaining vector were injected through the tail‐vein. Mice were sacrificed 3 weeks post injection.

### 2.5 Transverse aortic constriction (TAC)

Mice were anesthetized with a solution mixture of 95 mg kg^–1^ ketamine and 5 mg kg^–1^ xylazine administered via intraperitoneal injection. Mice were ventilated with a tidal volume of 0.2 mL and a respiratory rate of 110 breaths per minute (Harvard Apparatus). A longitudinal incision of 2–3 mm was made in the proximal sternum to allow visualization of the aortic arch. The transverse aortic arch was ligated between the innominate and left common carotid arteries with an overlaid 27‐gauge needle. The needle was then immediately removed, leaving a discrete region of constriction.

### 2.6 Luciferase assay

Luciferase assays were conducted by *CMV.Luc*, *SLN.Luc* and *CRM4.SLN.Luc* vectors in transfected HL1 cells and transducted mice (*n* = 4 per virus). HL1 cells were harvested 24 hours post transfection. Three weeks post injection, the atrium, ventricle, skeletal muscle, diaphragm, brain, lung, liver and kidney were harvested from each mouse. Samples were prepared with the Pierce Firefly Luciferase Glow Assay Kit (Thermo Scientific, Rockford, IL, USA) and quantified by BCA assay reagents (Thermo Scientific). Accordingly, 10 μg of total lysate was used for each sample and measured with the 1450 MicroBeta TriLux Microplate Scintillation and Luminescence Counter (Perkin Elmer, Shelton, CT, USA).

### 2.7 Fluorescence imaging

Mouse heart tissues were cut in a sagittal plane and fixed in 4% paraformaldehyde for 24 hours and moved to 20% sucrose for cryoprotection for 24 hours before being cryopreserved to OCT compound (Tissue‐Tek, Sakura Finetek, Torrance, CA, USA) at –80°C. Tissues were sectioned into 6‐μm thick slices (CM 3050S; Leica, Wetzlar, Germany). The slides were dehydrated in 100% ethanol for 10 minutes and dipped in two changes for xylene. Slides were sealed with glass slides using mounting medium with 4′,6‐diamidino‐2‐phenylindole (DAPI) (Vectashield, Burlingame, CA, USA) and analyzed using a confocal microscope (Observer.Z1; Zeiss, Oberkochen, Germany) in conjunction with Zen 2 Pro (Zeiss). With 200× magnification, 20 areas of the atrium and ventricle were randomly chosen per each sample with the same exposure time and correction and then one representative image was chosen randomly.

### 2.8 Western blot analysis

Tissue homogenates were lysed using RIPA buffer (Thermo Scientific) with protease inhibitor cocktail (Sigma‐Aldrich). Protein quantification was conducted using Pierce BCA Protein Assay Kit (Thermo Scientific). Proteins were resolved on 10% SDS‐PAGE gels followed by transfer to nitrocellulose membrane (Bio‐Rad, Munchen, Germany). Antibodies were raised against EGFP (Abcam, Cambridge, MA, USA), tubulin (Abcam) and sarcolipin (EMD Millipore, Burlington, MA, USA). Detection of the protein bands was performed in accordance with standard laboratory protocols using Pierce ECL Western Blotting Substrate (Thermo Scientific) with a ChemiDoc Touch Imaging System (Bio‐Rad, Hercules, CA, USA) and analyzed with Image Lab Software (Bio‐Rad, Hercules).

### 2.9 qRT‐PCR

RNA was isolated from tissue homogenates from the atrium, ventricle, skeletal muscle, diaphragm, brain, lung, liver and kidney from control, *CMV.EGFP*, *SLN.EGFP* and *CRM4.SLN.EGFP* groups using the RNeasy Mini Kit (Qiagen, Hilden, Germany). cDNA was synthesized using the Verso cDNA Synthesis Kit (Thermo Scientific) in accordance with the manufacturer's instructions. A qRT‐PCR was conducted on a BioRad CFX Connect Real‐Time System (Bio‐Rad, Hercules) using PerfeCTa SYBR Green FastMix, Low ROX (Quanta BioSciences, Beverly, MA, USA). EGFP expression fold was observed with the primers: forward 5′‐AATGAGAAAG GCAGAACCGGTGGATCCACCGGTCGC‐3′ and reverse 5′‐GATCAGCGAGCTCTAGTCGACCTTTA CTTGTACAGC‐3′. 18S rRNA was used for normalization with the primers: forward 5′‐TAACGAACGAGACTCTGGCAT‐3′ and reverse 5′‐CGGACATCTAAGGGCATCACAG‐3′.

### 2.10 Statistical analysis

Statistical analyses were performed using a two‐tailed Student's *t*‐test, with significant differences demonstrated as appropriate. Data are reported as the mean ± SD.

## 3 RESULTS

### 3.1 Characterization of sarcolipin in heart failure‐induced mice

**Figure 1:**
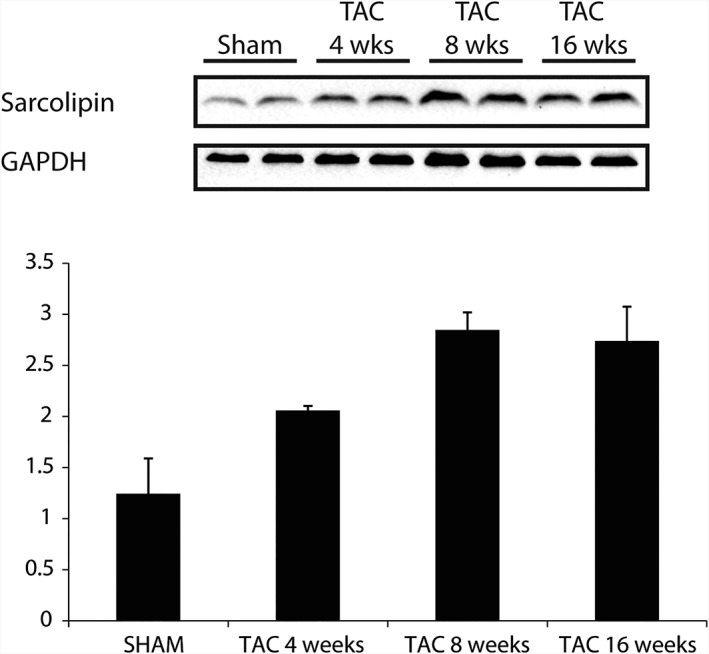
Sarcolipin expression increases in heart failure mouse samples. Sarcolipin expression was compared in heart failure mouse samples from 4, 8 and 16 weeks post TAC surgery

In heart failure sample mice models induced by TAC surgery, sarcolipin expression increased post TAC (Figure 1A). Post 8 weeks of TAC, sarcolipin levels were maintained.

### 3.2 Characterization of promoters *in vitro*

**Figure 2:**
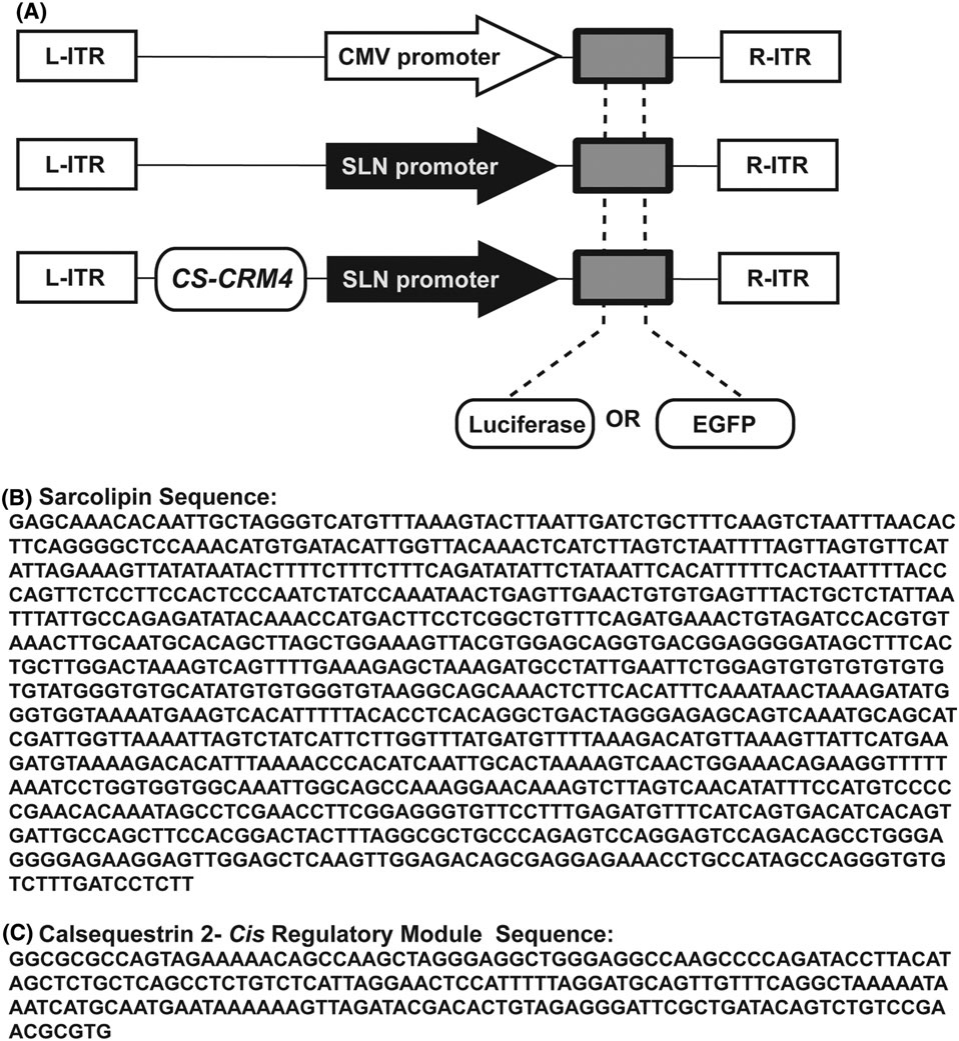
Generation of AAV constructs with various promoters. (A) Schematic depiction of CMV, SLN and *CRM4*.SLN promoters with luciferase or EGFP as a reporter gene. (B) The full 1289‐bp sarcolipin promoter sequence. (C) The full 207‐bp *CRM4* sequence

**Figure 3:**
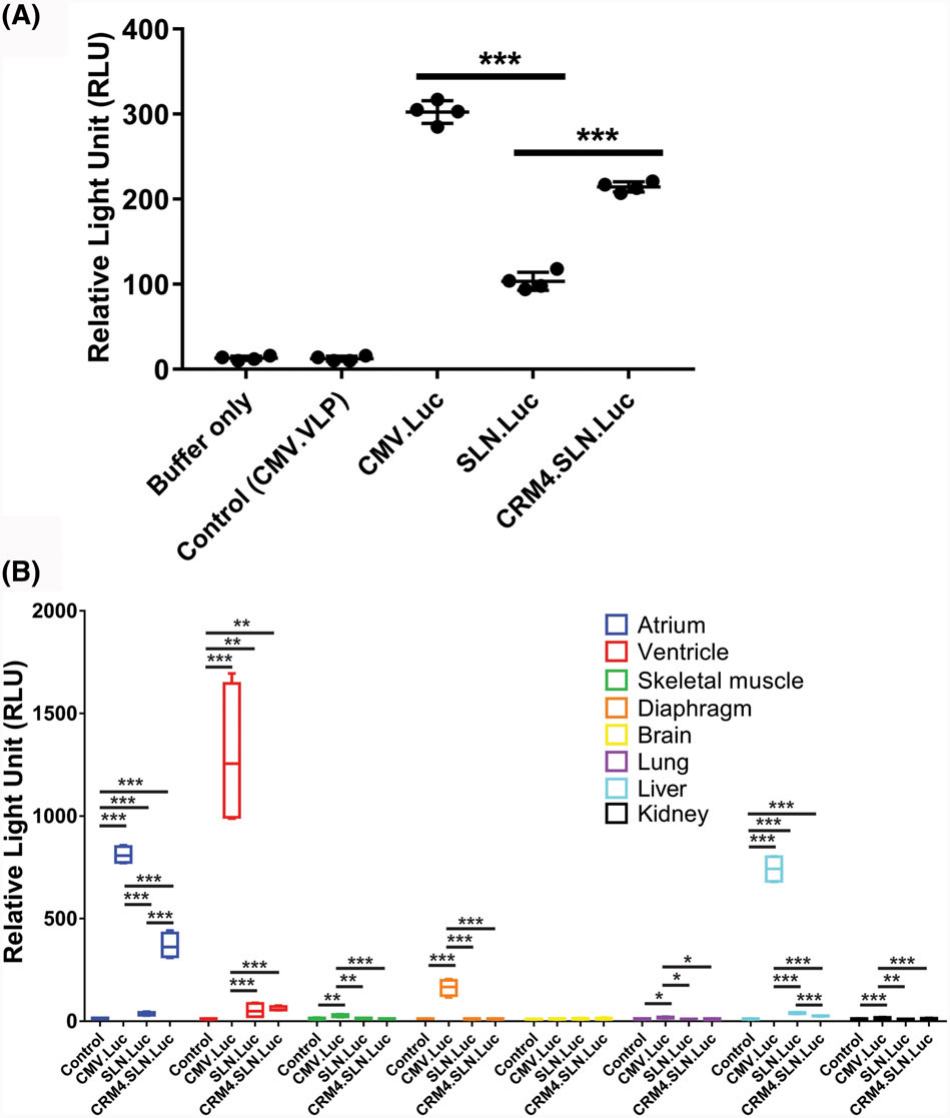
A luciferase assay confirmed *CRM4* enhanced‐atrial specificity and minimized off‐target expression. (A) A luciferase assay was conducted 24 hours post incubation in HL1 cells. Gene expression of control and luciferase containing CMV, SLN and *CRM4*.SLN vectors was measured. (B) *In vivo* gene expression was observed 3 weeks post tail vein delivery of control and luciferase containing CMV, SLN and *CRM4*.SLN vectors. A luciferase assay was conducted to determine bio‐distribution. Luciferase activity was normalized to background values. Statistical significance was measured with a two‐tailed Student's *t*‐test and significant differences are indicated: *p < 0.05, **p < 0.01, ***p < 0.005. Bar graphs represent the mean ± SD (*n* = 4)

Three different vectors containing luciferase were constructed: AAV9‐pTR‐CMV‐Luciferase (*CMV.Luc*), AAV9‐pTR‐SLN‐Luciferase (*SLN.Luc*) and AAV9‐pTR‐*CRM4*‐SLN‐Luciferase (*CRM4.SLN.Luc*) (Figure 2A). Relative promoter activities were evaluated *in vitro* in HL1 cells with viral constructs *CMV.Luc*, *SLN.Luc* and *CRM4.SLN.Luc* with a multiplicity of infection of 10^4^ vg cell^–1^. Cells infected with the control virus AAV9‐CMV‐VLP (*CMV.VLP*) showed only background activity. *SLN.Luc* had the lowest activity (8.28‐fold) followed by *CRM4.SLN.Luc* (16.38‐fold) and *CMV.Luc* (21.18‐fold) (Figure 3A). The *CRM4* sequence significantly improved SLN promoter activity (*p* < 0.005).

### 3.3 Biodistribution of vectors *in vivo*

#### 3.3.1 Luciferase

Three weeks post injection of *CMV.Luc* (*n* = 4), *SLN.Luc* (*n* = 4) and *CRM4.SLN.Luc* (*n* = 4), all mice, including control non‐injected mice (*n* = 4), were sacrificed. Bio‐distribution was observed in the atrium, ventricle, skeletal muscle, diaphragm, brain, lung, liver and kidney with the luciferase assay (Figure 3B). Control mice showed only background activity in all organs. *CMV.Luc* injected mice showed non‐specific distribution with significant activity in the atrium (*p* < 0.005), ventricle (p < 0.005), skeletal muscle (*p* < 0.01), diaphragm (*p* < 0.005), lung (*p* < 0.05), liver (*p* < 0.005) and kidney (*p* < 0.005) compared to control. *SLN.Luc* injected mice showed a distribution with significant activity in the atrium (*p* < 0.005), ventricle (*p* < 0.01) and liver (*p* < 0.005) compared to control. *CRM4.SLN.Luc* injected mice showed a distribution with significant activity in the atrium (*p* < 0.005), (*p* < 0.01) and liver (*p* < 0.005) compared to control. When *SLN.Luc* and *CRM4.SLN.Luc* were compared, there was a significant difference in the atrium and liver (*p* < 0.005). When *CMV.Luc* and *SLN.Luc* were compared, there was significant difference in the atrium (*p* < 0.005), ventricle (*p* < 0.005), skeletal muscle (*p* < 0.01), diaphragm (*p* < 0.005), lung (*p* < 0.05), liver (*p* < 0.005) and kidney (*p* < 0.005). When *CMV.Luc* and *CRM4.SLN.Luc* were compared, there was significant difference in the atrium (*p* < 0.005), ventricle (*p* < 0.005), skeletal muscle (*p* < 0.005), diaphragm (*p* < 0.005), lung (*p* < 0.05), liver (*p* < 0.005) and kidney (*p* < 0.01). The respective fluorescence measurements for each sample are reported in the Supporting information (Table S1).

#### 3.3.2 EGFP

The luciferase was exchanged with an EGFP reporter gene: AAV9‐pTR‐CMV‐EGFP (*CMV.EGFP)*, AAV9‐pTR‐SLN‐EGFP (*SLN.EGFP)* and AAV9‐pTR‐*CRM4*‐SLN‐EGFP (*CRM4.SLN.EGFP)*. Three weeks post injection of *CMV.EGFP* (*n* = 4), *SLN.EGFP* (n = 4) and *CRM4.SLN.EGFP* (n = 4), all mice, including control non‐injected mice (n = 4), were sacrificed.

##### Protein analysis

**Figure 4:**
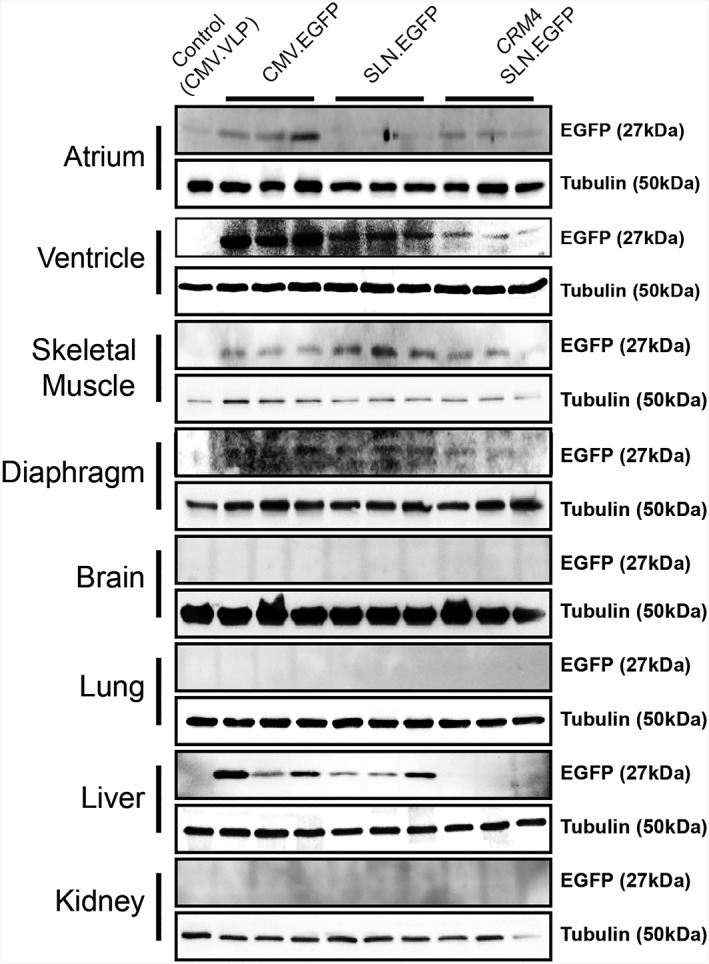
Atrial specificity and expression by *CRM4* were validated by an alternative reporter, EGFP. *In vivo* gene expression was observed 3 weeks post tail vein delivery of control and EGFP containing CMV, SLN and *CRM4*.SLN vectors. Western blot analysis was conducted to determine bio‐distribution with antibody EGFP and normalized with tubulin

Bio‐distribution was observed in the atrium, ventricle, skeletal muscle, diaphragm, brain, lung, liver and kidney with western blotting using 50 μg of protein (Figure 4). Control mice showed only background activity in all organs. EGFP was not detected in brain, lung and kidney samples. *CMV.EGFP* injected mice showed a distribution with expression in the atrium, ventricle, skeletal muscle, diaphragm and liver compared to control. *SLN.EGFP* injected mice showed EGFP expression in the atrium, ventricle, skeletal muscle, diaphragm and liver compared to control. *CRM4.SLN.EGFP* injected mice showed expression in the atrium, ventricle, skeletal muscle and diaphragm compared to control. Furthermore, dose‐dependent EGFP expression at 1 × 10^11^ vg or 5 × 10^11^ vg driven by *CMV* and *CRM4.SLN* promoters was observed. Three weeks post injection, all mice, including control non‐injected mice (*n* = 4), were sacrificed. Western blotting using 50 μg of protein was conducted in atrium, ventricle and liver with antibody EGFP and normalized with tubulin (see Supporting information, Figure S1). The atrium had comparable EGFP expression for 1 × 10^11^ vg or 5 × 10^11^ vg *CMV.EGFP* and dose dependency was observed in *CRM4.SLN.EGFP* injected samples. The ventricle and liver showed dose dependency for *CMV.EGFP* but not for *CRM4.SLN.EGFP* injected samples.

##### mRNA quantification

**Figure 5:**
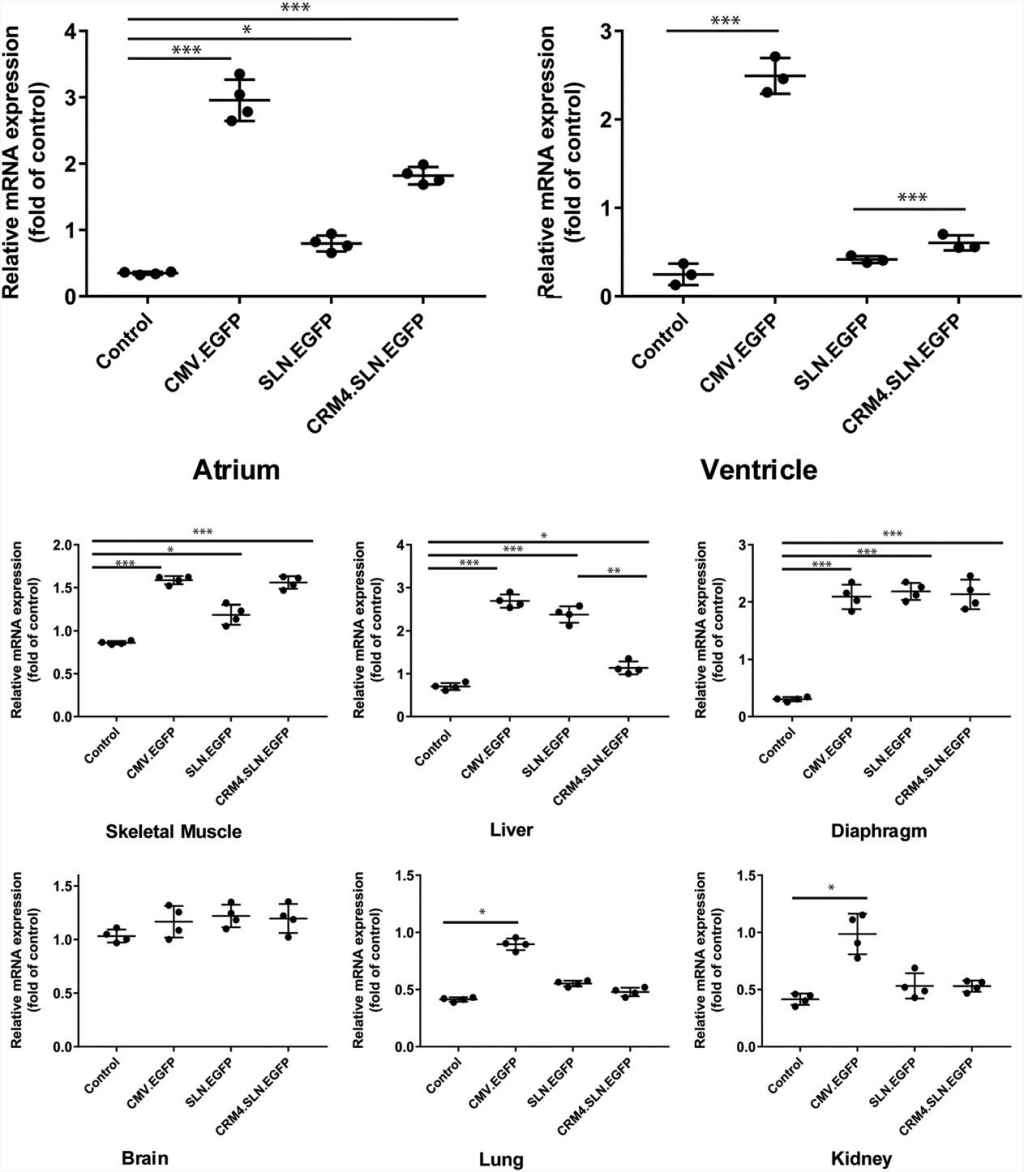
mRNA analysis validated EGFP gene expression profiles. *In vivo* gene expression was observed 3 weeks post tail vein delivery of control and EGFP containing CMV, SLN and *CRM4*.SLN vectors. qRT‐PCR analysis was conducted to determine bio‐distribution with EGFP and normalized with 18S. Statistical significance was measured with a two‐tailed Student's *t*‐test and significant differences are indicated: *p < 0.05, **p < 0.01, ***p < 0.005. Bar graphs represent the mean ± SD (*n* = 4)

Bio‐distribution was also observed with qRT‐PCR (Figure 5). Significant EGFP RNA expression was not observed in the brain. *CMV.EGFP* injected mice showed non‐specific distribution with significant expression in the atrium (8.3‐fold, *p* < 0.005), ventricle (10‐fold, *p* < 0.005), skeletal muscle (1.8‐fold, *p* < 0.005), diaphragm (6.6‐fold, p < 0.005), lung (2.2‐fold, *p* < 0.005), liver (3.8‐fold, *p* < 0.005) and kidney (2.3‐fold, *p* < 0.05) compared to control. *SLN.EGFP* injected mice showed a distribution with significant expression in the atrium (2.5‐fold, *p* < 0.05), skeletal muscle (1.3‐fold, *p* < 0.05), diaphragm (7.3‐fold, *p* < 0.005) and liver (3.4‐fold, *p* < 0.005) compared to control. *CRM4.SLN.EGFP* injected mice showed distribution with significant expression in the atrium (5.3‐fold, *p* < 0.005), skeletal muscle (1.8‐fold, *p* < 0.005), diaphragm (7.2‐fold, *p* < 0.005) and liver (1.6‐fold, *p* < 0.05) compared to control. When *SLN.EGFP* and *CRM4.SLN.EGFP* were compared, there was significant difference in the liver (*p* < 0.01). The atrium also shows decreasing trends.

Quantified luciferase values are shown in the table in supporting information (Table S1).

##### Fluorescence imaging

**Figure 6:**
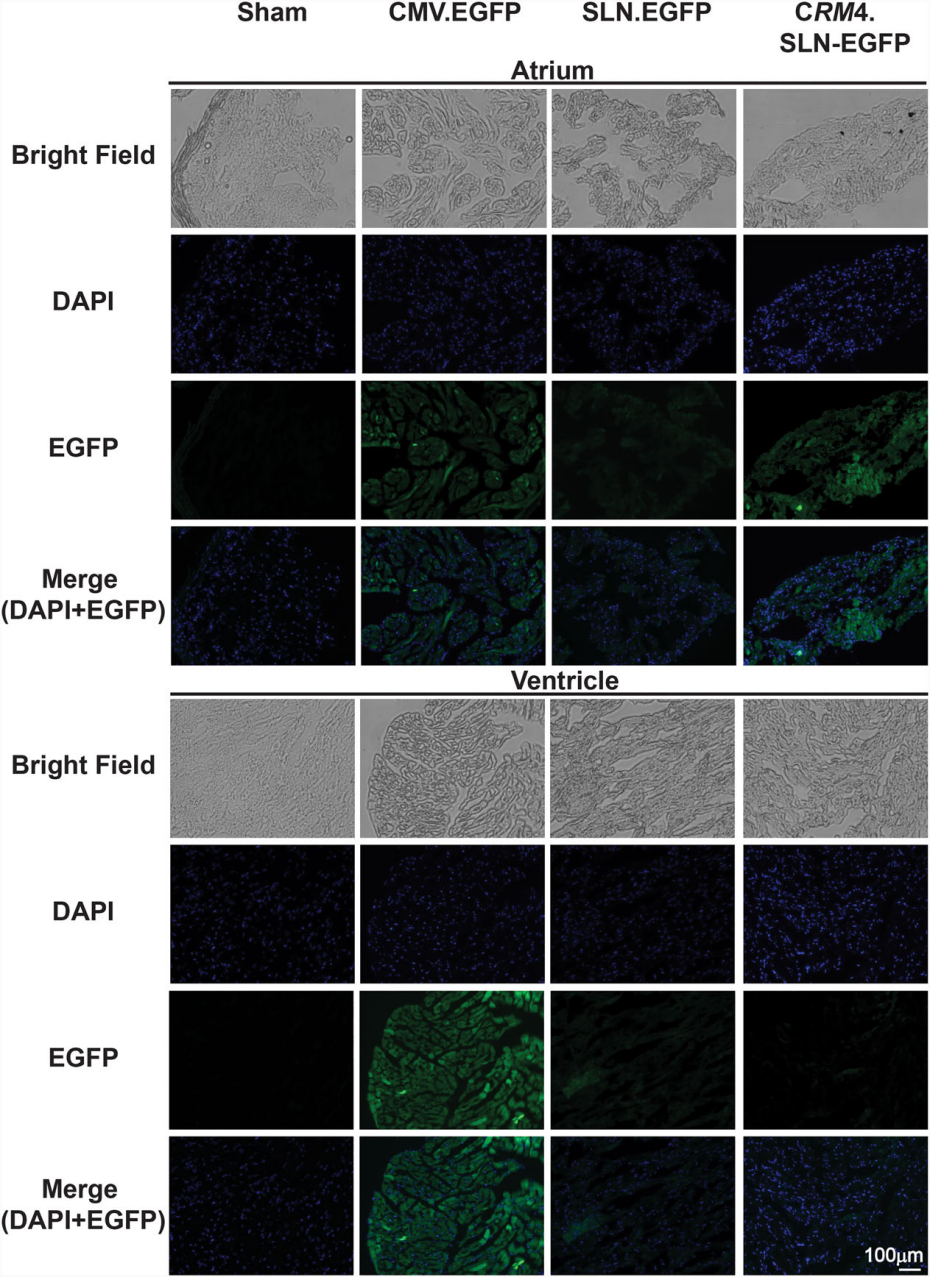
Visualization of biodistribution. *In vivo* gene expression in the atrium and ventricle of control and EGFP containing CMV, SLN and *CRM4*.SLN vectors was observed by fluorescence imaging using a confocal microscope at 200× magnification. Bright field, DAPI, EGFP and merged images were taken under same exposure and normalization settings

Furthermore, fluorescence imaging was conducted with atrium and ventricle tissue (Figure 6). Under the same exposure conditions, we observed the highest EGFP expression in both atrium and ventricle tissues of *CMV.EGFP* injected mice. In *SLN.EGFP* injected mice, EGFP expression was minimal in both the atrium and ventricle. On the other hand, in *CRM4.SLN.EGFP* injected mice, higher EGFP expression was observed in the atrium compared to the ventricle.

## 4 DISCUSSION

Currently, cardiac gene therapy has relied on adeno‐associated viruses as a main vector for gene transfer as a result of their high transduction efficiency.^25^ However, the promoter used to drive expression of the gene of interest often causes undesired activity in off‐target tissues, especially in hepatic tissue.^7^ Thus, cardiac‐specific promoters have been identified to improve cardiac specificity.^9^, ^10^, ^11^, ^12^, ^13^, ^14^, ^15^, ^16^ Even within these promoters, chamber specificity has been noted.^14^, ^15^, ^23^ Because the atrium and ventricle have individual functions, mechanisms and molecular signatures, pathophysiology varies in cardiac diseases as well. Thus, using a chamber‐specific promoter may be a better option than a global cardiac transgene expressing promoter. However, the transduction efficiency of chamber‐specific promoters is often compromised as a result of poor activity.^10^ With the current research on transcriptional regulation, we designed a combined a novel combination with synergistic effects to obtain enhanced atrial specificity.^21^ Various enhancer sequences have been reviewed,^26^, ^27^, ^28^, ^29^, ^30^ although *CRM4* was the most promising candidate as a result of its superior cardiac specificity. Thus, we have manipulated the promoter by adding *CRM4*, a cardiomyocyte‐specific enhancer, to improve transcriptional regulation of SLN.^21^

In the present study, we evaluated an atrial‐specific promoter sequence enhanced by a *cis*‐regulatory module *in vitro* and *in vivo*. Initially, to evaluate promoter efficiency, we observed expression in HL1 cells and confirmed that the *CRM4* within the gene cassette significantly improved luciferase activity driven by the SLN promoter in atrial cells (*p* < 0.005) (Figure 3A). Thus, we followed with *in vivo* bio‐distribution studies in wild‐type mice to quantify potential off‐target effects in six other tissues, including the skeletal muscle, diaphragm, brain, lung, liver and kidney. Skeletal muscle and diaphragm were investigated because high SLN expression was reported.^22^, ^23^ Other tissues were also measured to evaluate AAV9 off‐target effects.^5^, ^6^, ^7^ To compare gene expression levels under SLN and *CRM4*.*SLN* promoters, two reporter genes were used for assessment: luciferase and EGFP. The luciferase assay showed that *SLN.Luc* was not only expressed in the atrium, but also detected in the ventricle and liver. On the other hand, the *CRM4.SLN.Luc* showed highly selective luciferase activity in the atrium compared to the ventricle and other tissues. Strikingly, *CRM4* significantly minimized off‐target effects even compared to SLN promoter used alone.

For alternative verification for protein and mRNA analysis, EGFP was used as a supplementary reporter gene. Protein evaluation with western blotting supported the luciferase assay data, which showed that the *CRM4.SLN.EGFP* had exceptional atrial specificity and basal expression level in other tissues (Figure 4). The EGFP reporter gene offered additional insights by showing expression in skeletal muscle as well. Because SLN is also significantly expressed in skeletal muscle, expression was not unanticipated. However, in conjunction with the luciferase assay data, which showed expression in the atrium to be significantly stronger when all samples were compared in reference, skeletal expression was concluded to be lower than atrial expression. qRT‐PCR data of the EGFP transferred mice also supported atrial‐specific expression with *CRM4.SLN.EGFP* (Figure 5). Inconsistencies between RNA and protein levels in certain tissues such as the lung, kidney and brain reflect that our acquired data for qRT‐PCR showed relative values instead of actual mRNA. Furthermore, visualization of EGFP fluorescence was observed with sectioned tissue of the atrium and ventricle (Figure 6). The strong correlation between the luciferase and EGFP reporter genes both supported that the *CRM4.SLN* combination enhanced atrial specificity. Furthermore, we tested whether specificity to the atrium was preserved at higher doses of *CRM4.SLN.EGFP*. *CRM4.SLN.EGFP* showed unique dose dependency in the atrium. Although, at lower dosages, EGFP was previously not observed in the liver, at higher dosages, expression in the liver occurred. Nevertheless, *CRM4.SLN* confers exclusive atrial specificity among these three promoters.

For future application of this construct, the *CRM4.SLN* can be used for atrial‐specific gene therapy in disease models. Because sarcolipin expression is increased in heart failure states (Figure 1), the sarcolipin promoter in diseased states may be advantageous, similar to that of the ANF promoter.^11^ Furthermore, current therapeutic gene transfer for atrial fibrillation is highly limited, such as direct injection to the right and left atrium or to the atrioventricular node for rate control.^31^, ^32^ However, these approaches can lead to tissue damage and inflammatory response.^33^, ^34^ The most effective reported method is the gene painting method, which uses a poloxamer gel, dilute trypsin and vector mixture to increase contact time and penetration.^35^ However, this must be performed in open heart settings, which increases mortality risk.^36^ The use of this construct can offer a non‐invasive and technically simple approach for atrial‐specific gene therapy. The *CRM4* and SLN combination caused robust and highly‐specific atrial activity and is a promising method of targeting transcriptional mechanisms to improve atrial transgene transduction.

## CONFLICT OF INTEREST STATEMENT

The authors declare that they have no conflicts of interest.

## Supporting information


**Figure S1**. Dose‐dependent EGFP expression driven by CMV and *CRM4*.SLN promoters. *In vivo* gene expression was observed 3 weeks post tail vein delivery of control and EGFP containing CMV and *CRM4*.SLN vectors at doses 1E11 and 5e11 viral grams. Western blot analysis was conducted in the atrium, ventricle and liver with antibody EGFP and normalized with tubulin.
**Table S1**. Table of measured luminescence of luciferase assay biodistribution.
