# Supplementary material for: Enhancing atrial‐specific gene expression using a calsequestrin cis‐regulatory module 4 with a sarcolipin promoter
Source: J Gene Med. 2018 Dec 4;20(12):e3060. doi: 10.1002/jgm.3060 (PMC6519042; doi:10.1002/jgm.3060)
Supplement: Supplementary file 1 — Figure S1. Dose‐dependent EGFP expression driven by CMV and CRM4.SLN promoters. In vivo gene expression was observed 3 weeks post tail vein delivery of control and EGFP containing CMV and CRM4.SLN vectors at doses 1E11 and 5e11 viral grams. Western blot analysis was conducted in the atrium, ventricle and liver with antibody EGFP and normalized with tubulin. Table S1. Table of measured luminescence of luciferase assay biodistribution. [file JGM-20-e3060-s001.docx]

**Supporting Information**

**
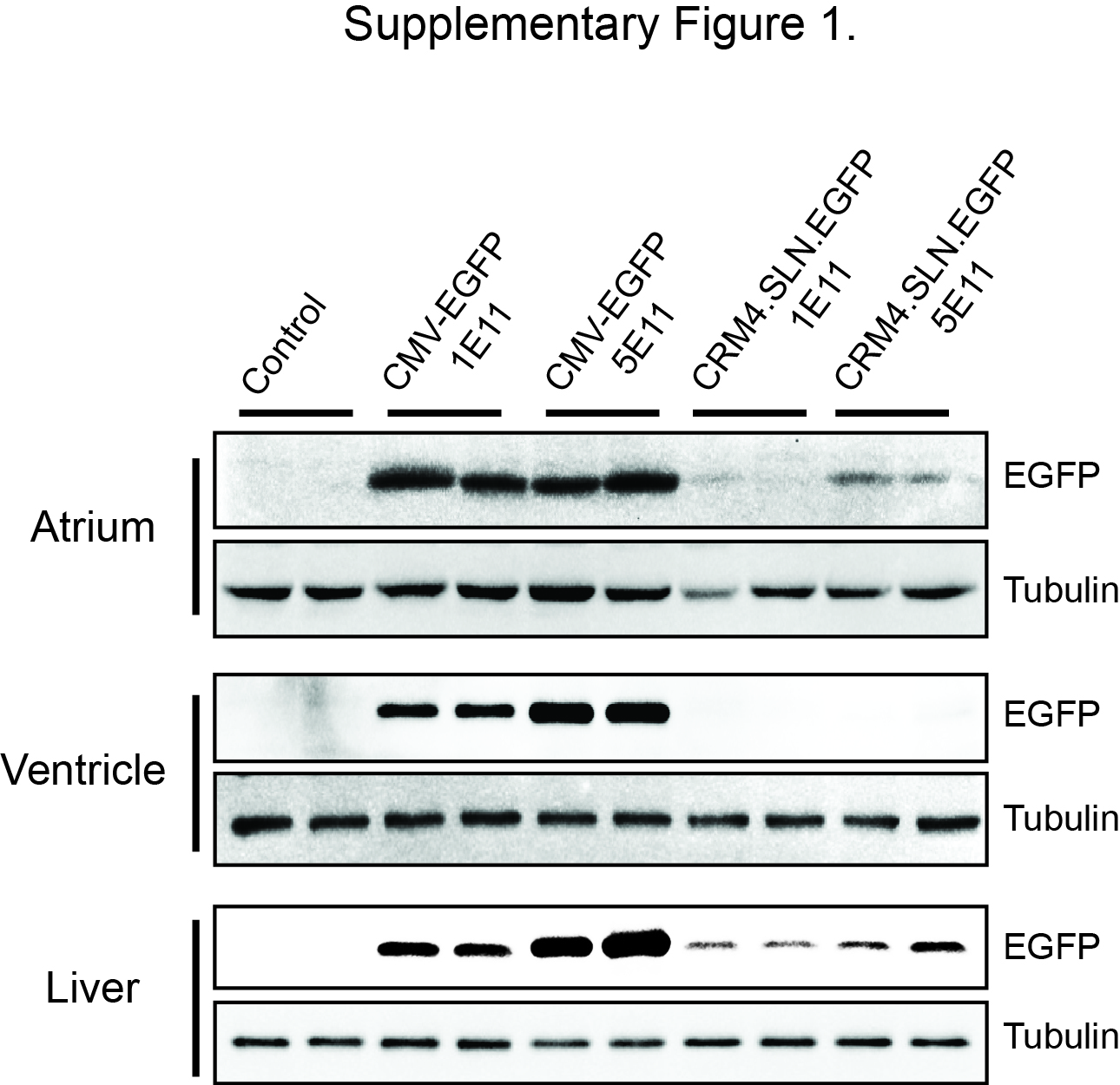
**

**Supplementary Figure 1.** **Dose dependent EGFP expression driven by CMV and *CRM4.*SLN promoters.** *In vivo* gene expression was observed 3 weeks post tail vein delivery of control and EGFP containing CMV and *CRM4*.SLN vectors at doses 1E11 and 5e11 viral grams. Western blot analysis was conducted in atrium, ventricle, and liver with antibody EGFP and normalized with tubulin.

**
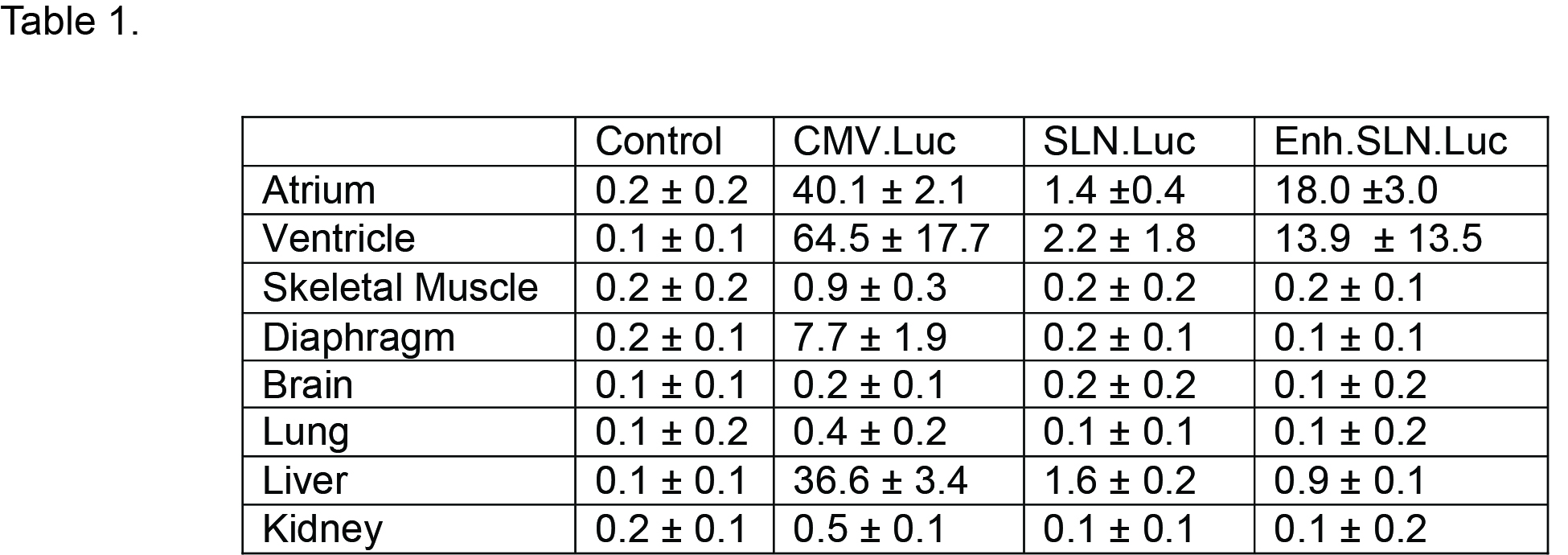
**

**Supplementary Table 1. Table of measured luminescence of luciferase assay biodistribution.** Table of the measured luminescence from the luciferase assay biodistribution from Figure 3.
